# Positive Human Immunodeficiency Virus (HIV) Test Following Influenza Vaccination: A Case Report

**DOI:** 10.7759/cureus.82928

**Published:** 2025-04-24

**Authors:** Rayyan Wazzi-Mkahal, Marianne Alwan, Najat I Joubran

**Affiliations:** 1 Nephrology, University of Toronto, Toronto, CAN; 2 Nephrology, Centre Hospitalier d'Auch, Auch, FRA; 3 Nephrology, Saint George Hospital University Medical Center, Beirut, LBN

**Keywords:** elisa, false-positive, hemodialysis, hiv, influenza

## Abstract

False-positive human immunodeficiency virus (HIV) test results following influenza vaccination are rare today. However, with evolving vaccine formulations, unexpected cross-reactivity remains a potential concern. This case highlights the importance of recognizing this phenomenon to prevent misdiagnosis and patient anxiety.

After obtaining her annual flu shot, an 82-year-old woman on hemodialysis (HD) was repeatedly discovered to have positive HIV enzyme-linked immunosorbent assay (ELISA) test results without having risk factors, and previously, the test was negative. As described in the literature, subsequent testing during the anticipated reversion period demonstrated a return to negativity, confirming a false-positive result.

Cross-reactivity between HIV ELISA tests and influenza vaccines has been reported infrequently due to potential immunologic interactions. This case shows the necessity of interpreting positive HIV results with caution, especially in recently vaccinated individuals and populations undergoing frequent serological testing, such as HD patients.

Clinicians should be aware of the cross-reactivity between the HIV ELISA test and the flu vaccination to recognize false-positive results, even with current influenza vaccine formulations. Awareness of this can prevent unnecessary patient distress, misdiagnosis, and unwarranted interventions.

## Introduction

Human immunodeficiency virus (HIV) testing is recommended at the start of hemodialysis (HD) and annually for patients undergoing HD, due to the elevated risk of blood-borne infections. This is supported by the Infectious Diseases Society of America (IDSA) guidelines, which highlight the role of routine testing, thereby reducing morbidity and mortality in high-risk groups [1]. Additionally, the Centers for Disease Control and Prevention (CDC) recommends routine HIV screening for all individuals aged 13-64 years in healthcare settings, with annual screening for vulnerable populations, which includes HD patients [2]. A notable example of the complexities surrounding HIV testing emerged in 1991-1992 during the influenza season when several false-positive serologic results for HIV and human T-lymphotropic virus 1 (HTLV-1) were observed following vaccination, as described by MacKenzie et al. [3]. Such findings emphasize the importance of HIV results in such a population receiving regular vaccinations. Herein, we report an HD patient who had an unexpected positive HIV test. 

## Case presentation

**Figure 1:**
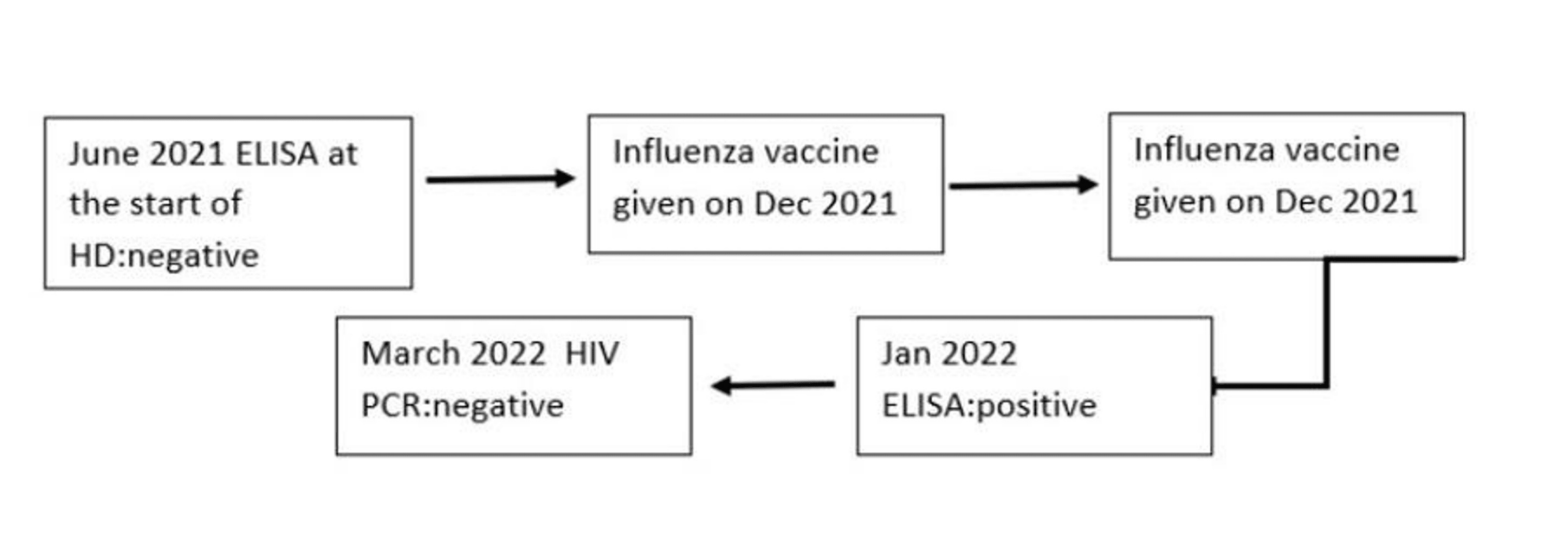
Case flowchart ELISA: enzyme-linked immunosorbent assay; HD: hemodialysis; HIV: human immunodeficiency virus; PCR: polymerase chain reaction

After obtaining consent, we report the case of an 82-year-old woman who has end-stage kidney disease (ESKD) on intermittent HD in one of the dialysis centers in Beirut, Lebanon. Her past medical history is otherwise significant for diabetes mellitus and dyslipidemia. When starting HD, the HIV enzyme-linked immunosorbent assay (ELISA) test was negative. However, seven months later, during routine testing at our facility and following national guidelines, she was unexpectedly found to have a positive HIV ELISA. The patient had never received a blood transfusion and was not sexually active. She was clinically stable without evidence of active infection: no weight loss, night sweats, fever, chills, or other systemic symptoms. She has no reported recent travel. The complete blood count was within range. Otherwise, her immunization history included a hepatitis B vaccine before initiating dialysis and yearly flu vaccination, the most recent of which was administered about 45 days before the detection. Her previous COVID-19 booster vaccine was given in September 2021. No illnesses, hospitalizations, or major interim events have occurred since the initiation of dialysis. Other possible causes of false-positive ELISA tests, such as autoimmune diseases, malignancy, or recent infections, were considered but were not supported by the clinical history or laboratory evaluation. An HIV polymerase chain reaction (PCR) was done 14 weeks after the initial positive test and revealed no viral replication. No HIV testing was performed between weeks 3 and 14, so the precise timing of seroconversion remains unknown (Figure 1).

## Discussion

The occurrence of HIV transmission after the initiation of HD is exceedingly uncommon, particularly in facilities that adhere to infection control protocols. The majority of HIV infections in dialysis patients are associated with pre-existing risk factors, such as intravenous drug use or blood transfusions, rather than the dialysis procedure itself [4]. In a multicenter study in the United States over one year, they found zero cases of nosocomial HIV transmission in 1,324 HD patients when standard infection control strategies were implemented [5]. Initial tests for the diagnosis of HIV infection include the Western blot and ELISA [1]. ELISA is frequently a low-cost screening technique and is more than 99% sensitive and specific [5]. A Western blot analysis is typically used when a false-positive enzyme immunoassay (EIA) result is suspected. However, it is estimated that 20% of patients will have an unclear or inconclusive result. If this happens, a PCR can be utilized to test for HIV-1 DNA and RNA [4]. Buffington et al. reported that up to 0.5% of blood donors had repeatedly reactive HIV screening tests by ELISA tests, which were not confirmed by immunoassays, indicating false-positive tests in low-prevalence populations [6]. False-positive HIV ELISA results have been documented in many settings, including autoimmune disorders, renal failure/HD patients, cystic fibrosis, numerous pregnancies or transfusions, hepatitis B infection, malaria, schistosomiasis, Q fever, rickettsial diseases, IVIg and rabies, and influenza vaccination [3,7]. A significant association between positive COVID-19 PCR tests and false-positive HIV tests has been observed [8]. It was reported in one study that 9% of chronic HD patients have positive HIV EIA and more than half either were false-positive or showed indeterminate Western blot patterns. Notably, 4.4% had persistent indeterminate Western blot test results despite no seroconversion over a five-year follow-up period, indicating the high prevalence of immune-mediated false-positive reactivity in this population. This is attributed to alloimmune responses from transfusions, previous transplant rejections, and circulating autoantibodies targeting gag antigens such as p24 and p55 [9]. Chou et al. conducted a cohort study of 404 chronic HD patients. They identified a false-positive rate of 0.5% when screening using ELISA, as two patients initially tested positive, but the Western blot analysis was negative [10]. While it seems low, this rate is relatively high in a low-prevalence population and carries significant clinical implications. Interestingly, a false-positive HIV antibody test was reported in two such patients, following therapy with alpha-interferon or due to circulating p-ANCA and/or myeloperoxidase, respectively [4]. Apart from renal failure and HD, our patient did not exhibit any risk factors and had an initial negative ELISA testing at the beginning of HD. A history of influenza vaccination exhibited a cross-reactivity with the HIV ELISA according to numerous investigations conducted in the 20th century [3,5,10]. In one study, 16 blood donors with positive HIV testing were followed. It seemed that many unverified viral EIA reactivities in blood donors were connected to the influenza vaccination in 1991-1992 [3]. Another study following blood donors in the same years revealed that multiple false-positive viral ELISA results were estimated to have occurred in 0.6-1.7% of blood donors who received the influenza vaccine [7]. Erickson et al. described a case of a 35-year-old man who developed a false-positive HIV EIA result 11 days after receiving an influenza vaccine. Western blot was negative, confirming vaccine-related cross-reactivity [11]. Similarly, Eguchi et al. described a case of a healthy woman who tested falsely positive for HIV by EIA following a recent influenza vaccination; however, the exact timing between the tests and the vaccination was not specified [12]. On the other hand, a case-control study published suggested that the test kits were most likely to blame for the cluster of numerous false-positive results in 1991 [7]. Johns Hopkins University's and Yale University's Department of Health considered influenza vaccination as a known cause of imprecise results on HIV antibodies by Western blot [11]. Several proposed mechanisms lead to false-positive ELISA tests following influenza vaccination. The cross-reactivity is due to non-specific immunoglobulin M (IgM) cross-reactivity, which may cross-react with antigens used in HIV EIA/ELISA tests [9]. The transient formation of immune complexes between vaccine antigen and the newly formed antibodies can lead to a false-positive result, especially in individuals with impaired immune complex clearance, such as HD patients. Another proposed mechanism involves the partial homology between influenza hemagglutinin and HIV-1 envelope transmembrane proteins (gp41/gp120), which share molecular similarity, thus resulting in the subsequent cross-binding of influenza vaccine-induced antibodies to HIV antigens [11,13]. There have been no reports in the literature indicating that newer vaccines cause false-positive ELISA results, which makes this case report unique. The duration of false-positive HIV ELISA reversion after an influenza vaccine was reported by one study by MacKenzie et al. in 1991-1992. ELISA samples were obtained from blood donors who had false-positive HIV results after receiving the influenza vaccine, occurring within 9-68 days post-vaccination, and they reported a reversion to HIV seronegativity within 52-130 days (mean: 75 days) [3]. No additional studies specifically addressing the duration of false-positive HIV ELISA reversion post-influenza vaccination were identified in the provided references. However, the study by MacKenzie et al. remains a key source of information on this phenomenon.

## Conclusions

Falsely reactive HIV test findings can have serious implications for individuals and healthcare professionals. Patients may experience psychological distress in addition to unnecessary investigations. This case highlights the critical importance of interpreting HIV serologic tests with caution in the HD population, particularly in the setting of recent influenza vaccination. Despite the use of relatively new vaccine formulations, cross-reactivity with ELISA remains a relevant phenomenon. Given that influenza vaccines are given annually, clinicians must remain vigilant to avoid misdiagnosis and treatment. This case highlights the need for increased awareness of this phenomenon and suggests future research to explore the persistence of vaccine-antibody interference in the vulnerable population. 

## References

[1] Lucas GM, Ross MJ, Stock PG (2014). Clinical practice guideline for the management of chronic kidney disease in patients infected with HIV: 2014 update by the HIV Medicine Association of the Infectious Diseases Society of America. Clin Infect Dis.

[2] Janssen RS (2007). Implementing HIV screening. Clin Infect Dis.

[3] MacKenzie WR, Davis JP, Peterson DE, Hibbard AJ, Becker G, Zarvan BS (1992). Multiple false-positive serologic tests for HIV, HTLV-1, and hepatitis C following influenza vaccination, 1991. JAMA.

[4] Mandayam S, Ahuja TS (2004). Dialyzing a patient with human immunodeficiency virus infection: what a nephrologist needs to know. Am J Nephrol.

[5] Marcus R, Favero MS, Banerjee S, Solomon SL, Bell DM, Jarvis WR, Martone WJ (1991). Prevalence and incidence of human immunodeficiency virus among patients undergoing long-term hemodialysis. The Cooperative Dialysis Study Group. Am J Med.

[6] Buffington J, Shapiro CN, Holman RC (1994). Multiple unconfirmed-reactive screening tests for viral antibodies among blood donors. Transfusion.

[7] Arnold NL, Slade BA, Jones MM, Popovsky MA (1994). Donor follow-up of influenza vaccine-related multiple viral enzyme immunoassay reactivity. Vox Sang.

[8] Gudipati S, Shallal A, Peterson E, Cook B, Markowitz N (2023). Increase in false-positive fourth-generation human immunodeficiency virus tests in patients with coronavirus disease 2019. Clin Infect Dis.

[9] Vardinon N, Yust I, Katz O, Iaina A, Katzir Z, Modai D, Burke M (1999). Anti-HIV indeterminate Western blot in dialysis patients: a long-term follow-up. Am J Kidney Dis.

[10] Chou CC, Sun CY, Wu MS (2007). Human immunodeficiency virus (HIV) infection screening in a dialysis unit. Ren Fail.

[11] Erickson CP, McNiff T, Klausner JD (2006). Influenza vaccination and false positive HIV results. N Engl J Med.

[12] Eguchi S, Takatsuki M, Soyama A, Torashima Y, Tsuji A, Kuroki T (2013). False positivity for the human immunodeficiency virus antibody after influenza vaccination in a living donor for liver transplantation. Liver Transpl.

[13] Copeland KM, Elliot AJ, Daniels RS (2005). Functional chimeras of human immunodeficiency virus type 1 Gp120 and influenza A virus (H3) hemagglutinin. J Virol.

